# Antifungal antibodies present in intravenous immunoglobulin derived from China

**DOI:** 10.1007/s42770-022-00894-z

**Published:** 2023-01-05

**Authors:** Yanfang Wang, Yugu Liu, Susu Jiang, Yan Zhao, Jianpiao Cai, Wei Hao, Ning Fu

**Affiliations:** 1 Innovation Platform for In Vitro Diagnosis of Guangdong Province, Zhujiang Hospital, Southern Medical University, Guangzhou, 510282 Guangdong China; 2 Microbiome Medicine Center, Department of Laboratory Medicine, Zhujiang Hospital, Southern Medical University, Guangzhou, 510282 Guangdong China; 3 State Key Laboratory of Emerging Infectious Diseases, Department of Microbiology, The University of Hong Kong, Hong Kong Special Administrative Region, Hong Kong, China

**Keywords:** IVIG, antifungal antibody, *Candida albicans*, *Aspergillus fumigatus*, *Cryptococcus neoformans*, *Talaromyces marneffei*

## Abstract

Fungal infections usually occur in immunocompromised patients. Intravenous immunoglobulin (IVIG) has been used as therapeutic interventions for many infectious diseases, but seldom applied in mycosis due to unknown antifungal specificity. This study aims to determine the presence of antifungal antibodies in IVIG. Binding reactivity of IVIG with crude and recombinant antigens of *Candida albicans, Aspergillus fumigatus*, *Cryptococcus neoformans* and *Talaromyces marneffei* were observed in a dose-dependent manner, similar with mixed normal human sera. The antifungal specificity was further confirmed by competitive enzyme-linked immunosorbent assays (ELISA) inhibited by rabbit specific antifungal polyclonal antibodies (PAbs) and homogenous crude antigens with inhibitions of 65.5-87.2% and 73.1-94.2%, respectively. Moreover, IVIG also reacted with fungal glycoproteins (Csa2*,* Cpl1 and Mp1p) in a dose-dependent way, which was inhibited by specific rabbit PAbs and homogenous antigens with different inhibitions and pulled down 72.8-83.8% of specific antibodies if preabsorption IVIG with Dynabeads® coupled with homogenous glycoproteins. These results furthermore verified the antifungal specificity of IVIG. Among four brands of IVIG, there was different antifungal IgG against *C. albicans* (*P* < 0.05) and *C. neoformans* (*P* < 0.05), while no difference for *A. fumigatus* (*P* = 0.086) and *T. marneffei* (*P* = 0.057). IVIG contained a significantly higher level of specific IgG for *C. albicans* than other three fungi (*P* <0.001). In conclusion, we proved antifungal IgG against *C. albicans*, *A. fumigatus*, *C. neoformans* and *T. marneffei* present in IVIG, which might be expected to provide a possible immunoregulation choice for mycosis and an evaluation to humoral immunity against fungi.

## Introduction

Fungal exposure has been associated with human fungal infections and allergic diseases. *Candida*, *Aspergillus* and *Cryptococcus* caused most of the fungal-related deaths in global fungal infections [1], while *T. marneffei* contributes unneglectable fungal-related death, especially for HIV-infected patients in Southeast Asia and southern China [2]. Besides limited therapeutic choices for fungal infections, host defense failed to clean fungi in the form of noninvasive colonization in immunocompromised individuals is an important contributing factor [3, 4]. Humoral response to opportunistic fungal infections is indispensable [5, 6], which is mediated by antibodies mainly reacted with various components of fungal cells, such as glycoproteins, polysaccharides and secondary metabolites. Antibody response induced by fungal glycoproteins were detected and used to diagnosis in mycosis patients [7, 8], while antifungal antibodies against polysaccharides such as beta-glucans, mannan and some crude antigens were sparkly reported in normal human sera [9–11].

Intravenous immunoglobulin (IVIG) is prepared from plasma pooled from thousands of healthy donors, which has been used in treatment of many diseases including infectious diseases, since IVIG has been proved with a diversity of antibacterial and antiviral specificities [12]. But the antifungal specificity was unknown. Pedraza-Sánchez S et al reported that IVIG therapy was beneficial to chronic oral candidiasis patients [13] by opsonization of *C. albicans* with polyvalent intravenous IgG and killing the fungi by blood leukocytes. This suggested IVIG as an optional adjuvant treatment for fungal infection. However, the direct evidence of presence and specificity of antifungal antibody in IVIG was absent. In normal human sera, antifungal antibodies against *C. albicans* and *A. fumigatus* in normal sera were reported [9, 11] However，these reports were just performed based on small number of serum samples, in which the antibody level showed a significant dispersion due to individual variation. A large number of serum samples could be needed to evaluate the antifungal antibodies in normal population.

In this study, we aimed to test antibody response to *C. albicans*, *A. fumigatus*, *C. neoformans* and *T. marneffei* by measuring antifungal IgG in different brands of IVIG derived from China. Antifungal IgG against recombinant protein Afmp1cr of *A. fumigatus*, Mp1p of *T. marneffei*, Csa2 of *C. albicans* and Cpl1 of *C. neoformans* were further tested to determine the possible target. It could be expected to evaluate antifungal humoral immunity in normal sera and to provide some evidences for antifungal therapy by IVIG.

## Materials and methods

### Fungi strains and crude fungal antigens

*T. marneffei* PM4 strain and *A. fumigatus* were obtained from the Department of Microbiology, University of Hong Kong. *C. neoformans* and *C. albicans* (BMU03833), which were originally clinical isolates recovered from infectious patients, were obtained from the Research Centre for Medical Mycology, Beijing University, China [14]**.** All the fungi were cultured in Sabouraud broth at 37°C for 3-7 days to prepare the crude antigens as reference described [14]. Briefly, fungal cells were collected and suspended in phosphate-buffered saline (PBS), after disruption by sonication, the supernatant was collected by centrifugation. The concentration of fungal antigens was determined by bicinchoninic acid (BCA) protein assay kit (Thermo Scientific).

### Expression of recombinant fungal glycoproteins in *Pichia pastoris*

Recombinant glycoproteins (Csa2 specific for *C. albicans* [15], Afmp1cr expressed as part of Afmp1p which specific for *A. fumigatus* [16, 17], Cpl1 specific for *C. neoformans* [18], Mp1p specific for *T. marneffei* [19]) were expressed previously in *Pichia pastoris* with his-tag. In brief, according to the manufacturer's instructions, the truncated genes of recombinant fungal glycoproteins were expressed in *P. pastoris* strain GS115 (Invitrogen). Nitrilotriacetic acid affinity chromatography (Qiagen, Hilden, Germany) was used to purify the recombinant protein, after a large-scale production of the protein. The Bicinchoninic Acid Protein Assay Kit (Sigma-Aldrich, St. Louis, MO) was used to measure the concentration of purified fungal glycoproteins according to the manufacturer’s instructions.

### Preparation of rabbit antifungal polyclonal antibodies (PAbs)

In previously study, we also prepared rabbit PAbs against crude fungal antigens of *C. albicans* (anti-*C. albicans*), *A. fumigatus* (anti-*A. fumigatus*) and recombinant glycoproteins including anti-Csa2, anti-Afmp1cr, anti-Cpl1 and anti-Mp1p PAbs [14, 15, 18, 19]. These PAbs were purified from immune rabbit serum by ammonium sulfate precipitation. Normal rabbit IgG from Sigma-Aldrich was used as control.

The potency of antifungal antibody in rabbit PAbs were tested by indirect enzyme-linked immunosorbent assay (ELISA), which was set to be the concentration when the ratio of A_450 antifung al rabbit PAbs_ /A_450 normal rabbit IgG_ > 2.1. The microplates were coated with crude antigens and recombinant proteins (Csa2, Afmp1cr, Cpl1 and Mp1p) followed by blocking and washing, then aliquots of 50μl/well of rabbit antifungal specific PAbs or normal rabbit IgG (in serial four-fold dilution) was added into microplate at 37°C for 1 h. After washing, a HRP labeled goat anti-rabbit IgG antibody (Sigma-Aldrich) at a working concentration of 1:2000 was added and incubated at 37°C for 30 min，color developing reaction was stopped and the absorbance was read at 450 nm.

### Normal human ser

Normal serum samples from 563 healthy donors, which were detected with Mp1p antigen negative in previous study [20], were mixed and stored at -80°C, which was approved by the Ethics Committee of the Zhujiang Hospital of Southern Medical University (No. ZJYY-2011-JYYXB-002). The concentration of total IgG in mixed human sera was measured by BNPro Spec® System (Dade Behring, Siemens) in a concentration of 14.5mg/ml.

### Commercial IVIG and Human Serum Albumin (HSA)

IVIG preparations from four brands were measured. The IVIG preparations of Taibang® human immune globulin intravenous (Shandong Taibang, Inc., China), Nanyue® human immune globulin intravenous (Nanyue Biopharming Co. Ltd., China), and RAAS® human immune globulin intravenous (Shanghai RAAS Blood Products Co. Ltd., China) were purified by cold ethanol fractionation. They were formulated as a liquid with a low pH (pH 4) in the presence of maltose as a stabilizer and contained 50 mg/ml IgG. Intragam® P human normal immunoglobulin (CSL, Hongkong, China) was made by chromatographic fractionation of large pools of human plasma donated by Hong Kong’s voluntary and non-remunerated donors, which contained 60 mg/mL IgG (PH 4.25). Human Serum Albumin (HSA, 250mg/ml) (Grifols Biologicals LLC, CA, USA) from plasma of healthy donors was used as control.

### Measurement of antifungal antibodies in IVIG and mixed normal human sera by indirect ELISA

Crude antigens and recombinant proteins (Csa2, Afmp1cr, Cpl1 and Mp1p) of four fungi at a concentration of 0.25-1μg/ml were coated into microplates (plant bottom, KE-96-12, YJM LABWARE CO. Ltd, China), followed by blocking with 0.25% casein at 4°C for 24 h. Then aliquots of 100μl/well of IVIG (from 1250 μg/ml in serial two-folds diluted) was added to microplate at 37°C for 1h, after washed with 0.5%Tween-20 PBS, the goat anti-human IgG antibody conjugated with HRP (Sigma-Aldrich, St Louis, MO, USA) at a working concentration of 1:3000 was added at 37°C for 30 min followed by washing, substrate solution (TMB) was added at room temperature for 10 min. The reaction was stopped by the addition of 1M H_2_SO_4_, and absorbance at 450 nm(A_450_) was measured by an ELISA reader. The absorbance values of IVIG in different concentrations were detected. Antifungal IgG levels against crude fungal antigens in different IVIG preparations were calculated as the value of Signal-to-Cutoff (S/CO) ratio [21] (described in Results Part). Mixed normal human sera from 563 healthy donors in serial 10-fold dilution were also measured for the antifungal IgG as above.

### Confirmation for specificity of antifungal IgG by competitive ELISA with rabbit specific PAbs or fungal antigens

Briefly, rabbit specific PAbs against homogenous fungal antigens were used as inhibitors from 2500μg/ml in a two-fold dilution, and normal rabbit IgG as negative control, which were mixed with IVIG and added to microplates coated with fungal antigens, respectively, then followed by adding HRP labeled goat-anti human IgG. The colors development reaction was the same as above indirect ELISA. The competitive test was repeated in three individual experiments.

Additionally, fungal antigens in different concentrations were also used as inhibitors to confirm the specificity of antifungal antibodies in IVIG. Serial diluted inhibiting antigens, including crude antigens and recombinant proteins of *C. albicans*, *A. fumigatus*, *C. neoformans* and *T. marneffei* were mixed and incubated with Taibang® IVIG at 4°C for 16 h, respectively. Then the mixtures were detected by ELISA as above with microplates coated with fungal antigens of *C. albicans*, *A. fumigatus*, *C. neoformans* and *T. marneffei*. The inhibition test was repeated in three individual experiments. The inhibition was calculated as below: Inhibition = (A_450 without inhibitor_ - A_450 inhibitor_) / A_450 without inhibitor_×100%.

### Antibody absorption test by magnetic Dynabeads coupled with His-tagged recombinant fungal glycoproteins

His-tagged recombinant fungal glycoproteins (Csa2, Afmp1cr, Cpl1 and Mp1p) were coupled to magnetic Dynabeads, according to manual procedures of commercial Dynabeads® His-Tag Isolation & Pulldown (Life Technologies AS, Norway). BCA protein assay was used to determine the concentration of recombinant proteins before and after coupling procedures, until it reached a claimed capacity of 40μg His-tagged protein per mg of Dynabeads®. Dynabeads coupled with recombinant proteins were mixed and incubated with 1250 μg/ml Taibang® IVIG at 4°C for 16 h, then the mixture was added to microplates coated the same recombinant protein, followed by conventional indirect ELISA. The competition efficiency of Dynabeads coupled with His-tagged proteins was calculated by the formula as below: Competition efficiency = (A_450 before incubation_- A_450 after incubation_) / A_450 before incubation_×100%.

### Statistical analysis

Antifungal IgG levels in S/CO ratio values among different brands of IVIG were compared with nonparametric Kruskal-Wallis test. A value of *p* <0.05 was considered significant. Antifungal IgG levels against different fungi were compared with independent samples t test. A two-sided *p* value of < 0.05 was considered significant. All statistical analyses were performed using SPSS (version 23.0).

## Results

### Antifungal IgG against *C. albicans*, *A. fumigatus*, *C. neoformans* and *T. marneffei* were observed in mixed normal human sera and IVIG

**Fig. 1:**
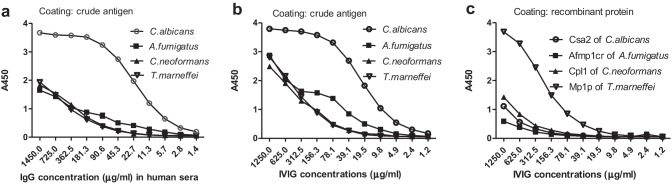
The binding of fungal antigens with normal human sera and IVIG. Mixture of normal human sera collected from 563 healthy donors in Guangdong Province reacted with fungal crude antigens (**a**). The binding of Taibang® IVIG with crude antigens (**b**) and recombinant proteins(**c)** from *C. albicans*, *A. fumigatus*, *C. neoformans* and *T. marneffei*

Similar antifungal IgG reactivity patterns in both mixed normal human sera and Taibang® IVIG were observed with crude antigens from *C. albicans*, *A. fumigatus*, *C. neoformans* and *T. marneffei* (Fig. 1a, b). Strongest positive signal was showed in crude antigens of *C. albicans* with mixture of normal human sera from 563 healthy donors and IVIG. Lower A_450_ values was detected for *A. fumigatus*, *C. neoformans* and *T. marneffei*. This indicated a possibility of different levels of antifungal IgG in sera from different population.

To further confirm the presence of antifungal IgG, recombinant fungal glycoproteins were used to verify the binding of IVIG with fungus. The results showed that a different reaction pattern of IVIG and recombinant proteins was observed, with the highest positive signal with Mp1p from *T. marneffei*. It confirmed the possibility of antifungal antibodies in IVIG for *C. albicans*, *A. fumigatus*, *C. neoformans* and *T. marneffei*, and reminded us that the complexity of fungal antigens would influence the reaction pattern. Taibang® IVIG reacted with recombinant proteins (Csa2, Afmp1cr and Cpl1) in lower A_450_ values than homogenously crude antigens, while Mp1p with a highest positive reaction than crude antigen (Fig. 1c). This may hint that Mp1p could provide more targeting epitopes of antifungal IgG than other recombinant proteins.

### Specificity of antifungal IgG by competitive ELISA using rabbit antifungal PAbs

**Fig. 2:**
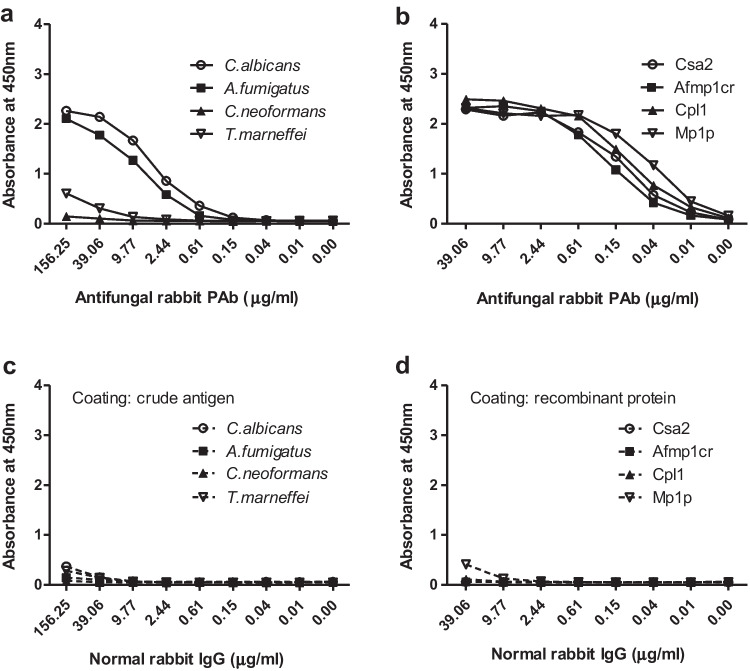
Determination the potency of antifungal specific PAbs compared with normal rabbit IgG. The crude fungal antigens reacted strongly with specific antifungal rabbit PAbs (anti-*C. albicans* and anti-*A. fumigatus*) and weakly reacted with anti-Cpl1 PAb and anti-Mp1p PAbs (**a)**. Weaker reaction was observed in normal rabbit IgG of a concentration of 156 μg/ml with crude antigen (**c**). Higher potency was found for specific antifungal rabbit PAbs (anti-Csa2, anti-Afmp1cr, anti-Cpl1 and anti-Mp1p PAbs) with recombinant fungal proteins (**b**) than that with crude antigens (**a**), while no reaction was observed with recombinant fungal proteins (Csa2, Afmp1cr and Cpl1) except a paint positive with Mp1p in a concentration of 39 μg/ml for normal rabbit IgG (**d**)

Competitive ELISA was conducted to confirm the antifungal specificity of IVIG using rabbit PAbs against homogenously fungal antigens as inhibitors. First, we evaluated the potency of rabbit PAbs against crude and recombinant antigens comparing with normal rabbit IgG. The binding potency concentrations of rabbit specific PAbs to crude antigens were 0.15μg/ml and 0.61μg/ml for anti-*C. albicans* and anti-*A. fumigatus,* respectively (Fig. 2a, c). The binding potency of PAbs against recombinant proteins reacted strongly with corresponding proteins with the binding concentration of 0.01μg/ml for anti-Csa2 PAb, anti-Afmp1cr PAb, anti-Cpl1 PAb and 2.4ng/ml for anti-Mp1p PAb, respectively (Fig. 2b, d). Due to absent of PAbs specific for crude antigen of *C. neoformans* and *T. marneffei*, we used anti-Cpl1 and anti-Mp1p PAbs which reacted with a limit detection of 312.5μg/ml and 9.77μg/ml for crude antigens of *C. neoformans* and *T. marneffei,* respectively.

**Fig. 3:**
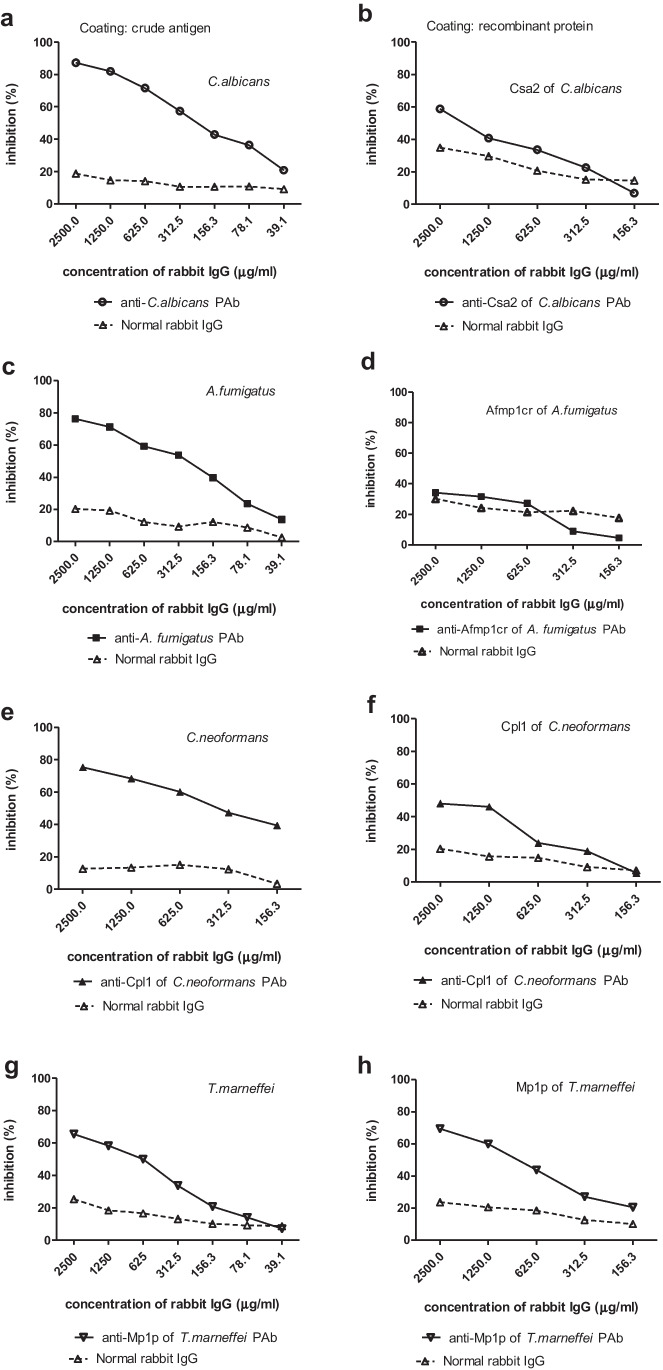
The binding of crude and recombinant fungal antigens with Taibang® IVIG was inhibited by fungal specific PAbs in competitive ELISA. Rabbit anti-*C. albicans* PAbs inhibited the binding of IVIG with *C. albicans* crude antigen (**a**). Rabbit anti-Csa2 PAbs inhibited the binding of IVIG with recombinant Csa2 (**b**). Rabbit anti-*A. fumigatus* PAbs inhibited binding of IVIG with *A. fumigatus* crude antigen (**c**). Anti-Afmp1cr PAbs inhibited the binding of IVIG with Afmp1cr. **e** Rabbit anti-Cpl1 PAbs inhibited the binding of IVIG with *C. neoformans* crude antigen (**d**) and recombinant Cpl1 (**f**). Rabbit anti-Mp1p PAbs inhibited the binding of IVIG with Binding of IVIG with *T. marneffei* crude antigen (**g**) and recombinant Mp1p (**h**). Normal rabbit IgG from Sigma-Aldrich was used as control. The inhibition test was repeated in three individual experiments

The binding of fungal crude antigens with IVIG was obviously inhibited by corresponding rabbit anti-*C. albicans*, anti-*A. fumigatus*, anti-Cpl1 and anti-Mp1p PAbs respectively in a dose-dependent way (Fig. 3a, c, e, g), which confirmed the antifungal specificity of IVIG. However, rabbit specific PAbs (Fig. 3b, d, f) showed lower inhibitions on binding of IVIG with most of recombinant proteins except for Mp1p, even though with high potency. The binding of IVIG with crude antigens was significantly inhibited by anti-*C. albicans* PAb compared with normal control (87.2% vs 18.8%) (Fig. 3a), while a lower inhibition of rabbit anti-Csa2 PAb and normal control (58.8% vs 34.9%) was observed in the binding activity of IVIG with Csa2 (Fig. 3b). Rabbit anti-*A. fumigatus* PAb obviously inhibited IVIG binding with crude antigen (76.3% vs 20.3%, Fig. 3c), while no difference on inhibitions between anti-Afmp1cr PAb and normal control (34.9% vs 30.1%, Fig. 3d). For *C. neoformans* and *T. marneffei*, we only prepared PAbs for recombinant proteins, which were used as inhibitors for IVIG binding with both crude and recombinant antigens. The results showed that both anti-Cpl1 and anti-Mp1p PAbs could significantly inhibit the binding of IVIG with fungal crude antigens (75.3% vs 12.63% for *C. neoformans*; 65.5% vs 25.3% for *T. marneffei*) and recombinant antigens than normal control (48.0% vs 20.3% for Cpl1; 69.5% vs 23.7% for Mp1p) (Fig. 3e - h). These observations demonstrated that antifungal antibodies against the recombinant proteins was only small a part of antifungal IgG against for the four fungi. Normal rabbit IgG also showed a weak inhibition in a dose dependent manner, which indicated a possibility of containing low level of antifungal antibody in normal rabbit IgG.

### Specificity of antifungal IgG by competitive ELISA using the same crude fungal antigens

**Fig. 4:**
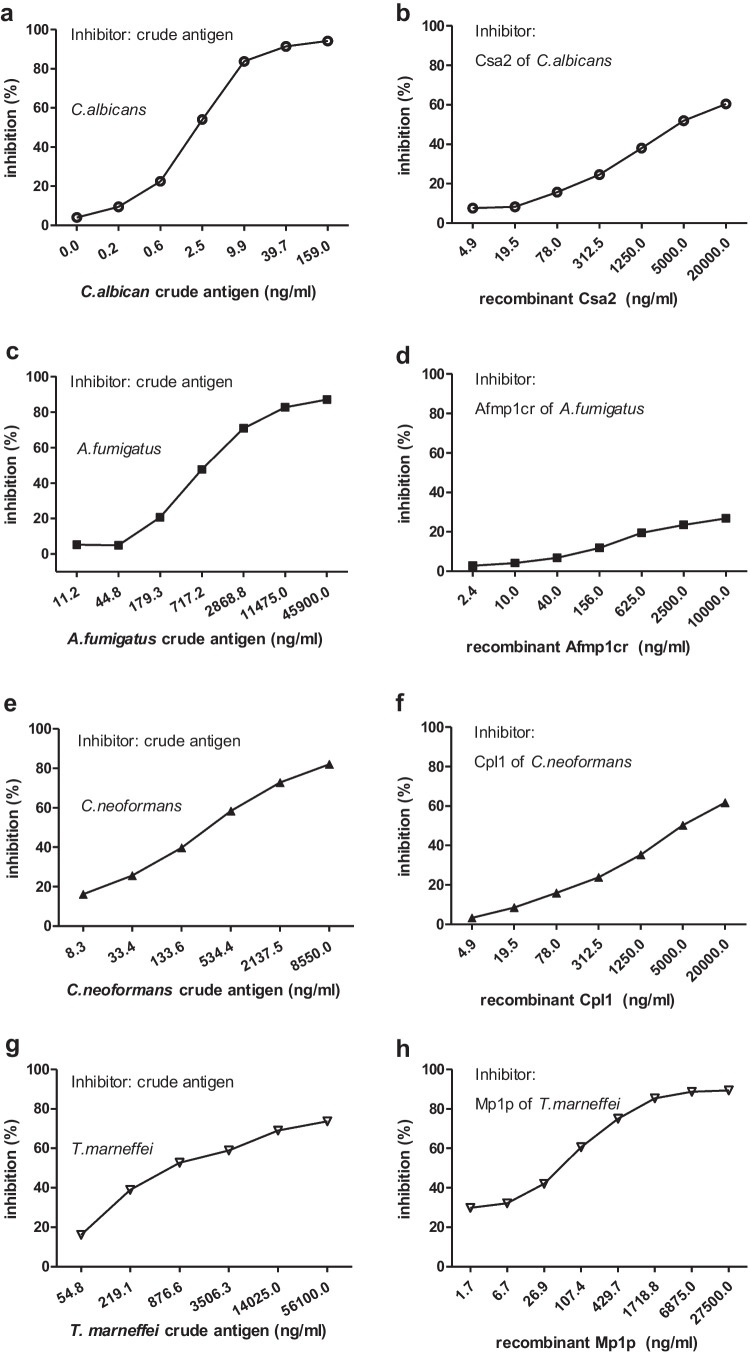
The binding of fungal antigens with Taibang® IVIG was inhibited by homogenous antigens in competitive ELISA. The binding of coated fungal crude and recombinant antigens with IVIG was inhibited by homogenous crude and recombinant antigens of *C. albicans* (**a**, **b**), *A. fumigatus* (**c**, **d**), *C. neoformans* (**e**, **f**) and *T. marneffei* (**g**, **h**),which preincubated with IVIG at 4°C for 16h, respectively. The inhibition test was repeated in three individual experiments

For further confirmation of antifungal specificity of IVIG, the same crude antigen was used as inhibitor. The results showed that crude antigens could effectively inhibit IVIG binding to microplates coated crude antigens. The half maximal inhibitory concentration (IC 50) was estimated to be 2.02ng/ml, 670.80 ng/ml, 258.60 ng/ml and 261.90 ng/ml for crude antigens of *C. albicans*, *A. fumigatus*, *C. neoformans* and *T. marneffei*, respectively (Fig. 4). All the inhibitive effect by crude antigens was in dose-dependent, which suggested that the antibodies towards fungal antigens were specific. These results were also consistent with inhibition results by antifungal PAbs, which further confirmed the presence of antifungal antibody in IVIG, especially for *C. albicans* with the lowest IC 50.

### Specificity of antifungal IgG against recombinant fungal glycoproteins

**Fig. 5:**
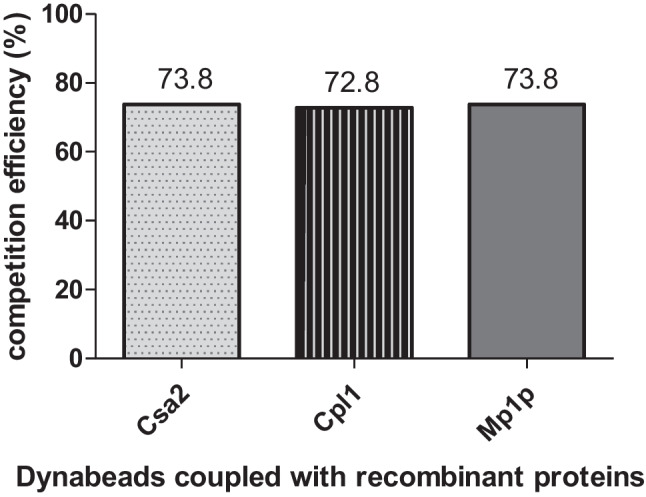
Specific binding of antifungal antibodies in IVIG with his-tagged recombinant proteins. IVIG was mixed and incubated with Dynabeads coupled with his-tagged recombinant fungal proteins (40μg) at 4°C for 16 hours, followed by reaction with microplates coated with corresponding recombinant proteins (Csa2, Cpl1 and Mp1p), and A_450_ was determined before and after incubation . The competition efficacy was calculated as the formula: competition efficiency = (A_450 before incubation_ - A_450 after incubation_) / A_450 before incubation_×100%

Additional competitive ELISA was also conducted to compete binding of IVIG with four recombinant fungal proteins coated on microplate, each of which shown a distinct degree of inhibition. The half maximal IC 50 was estimated to be 1107 ng/ml, 4517 ng/ml and 92.01 ng/ml for recombinant antigens Csa2 of *C. albicans*, Cpl1 of *C. neoformans* and Mp1p of *T. marneffei* respectively (Fig. 4). For Afmp1cr of *A. fumigatus，*no IC 50 was determined despite a 100-fold inhibitor dose of coated antigen*.* A highest inhibition of 89.40% was observed, when Mp1p was used as inhibitor (Fig. 4h), with an estimated IC50 of 92.01ng/ml. This result was consistent with the result of anti-Mp1p PAb inhibiting test (Fig. 3h) as well as antibody absorption test (Fig. 5), which confirmed the specificity of reaction.

For further confirming the specificity of antifungal antibodies, IVIG was absorbed with Dynabeads coupled with recombinant proteins (40μg) for each fungus, 72.8%-73.8% of IVIG decreased when tested with recombinant proteins (Csa2, Cpl1 and Mp1p) coated on microplates (Fig. 5). These results further proved the binding specificity of IVIG with *C. albicans*, *C. neoformans* and *T. marneffei*. Unfortunately, we could not obtain the result about Afmp1cr since we failed to couple Afmp1cr to Dynabeads.

### Different levels of antifungal IgG in four manufactures of IVIG preparations

**Fig. 6:**
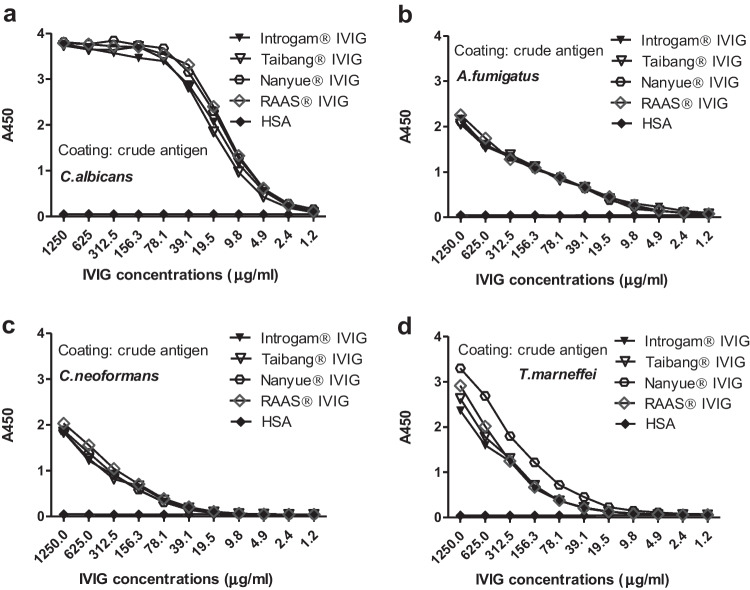
The binding of crude fungal antigens and antifungal IgG in four brands of IVIG. Four brands of IVIG in serial diluted reacted with crude antigens of *C. albicans* (**a**), *A. fumigatus* (**b**), *C. neoformans* (**c**) and *T. marneffei* (**d**). HSA was set as a negative control. IVIG contained significantly higher level of specific IgG for *C. albicans* than for other three fungi (*P* < 0.001) with the lowest for *C. neoformans.* No difference of the specific IgG level was observed between antifungal IgG specific *A. fumigatus* and *T. marneffei* (*P* = 0.772)

Similar reaction curves between increasing IVIG concentrations with crude antigens of *C. albicans, A. fumigatus, C. neoformans* and *T. marneffei* was observed among different brands of IVIG (Fig. 6), with the highest positive signal for *C. albicans*, which was consistent with normal human sera. No binding of HSA with IVIG was observed. S/CO ratio value was used to express the antifungal IgG level in 1mg/ml IVIG. The S/CO ratio was described as A_450 IVIG_ / A_450 HAS_ then divided by the concentration of IVIG and multiplied by 1000 to be expressed the ratio in 1 mg/ml IVIG. The A_450_ values were chose in a linear range in which the concentration of IVIG for *C. albicans, A. fumigatus*, *C. neoformans* and *T. marneffei* were 19.53μg/ml, 312.5μg/ml, 312.5μg/ml and 312.5μg/ml, respectively to calculate the S/CO ratio value. The S/CO ratio value would be represented the level of the antifungal IgG in 1 mg/ml IVIG, which were showed in Table 1 in mean ± standard deviation.

**Table 1:** Antifungal IgG level against crude fungal antigens in IVIG from four brands

Crude antigens	Signal-to-Cutoff (S/CO) ratio ^a^				*P* value
	RAAS® IVIG	Nanyue® IVIG	Taibang® IVIG	Intragam®IVIG	
*C. albicans*	***2379.80±33.89 ***^***b***^	2282.01±33.41	1833.08±47.91	2054.74±48.55	***0.016***
*A. fumigatus*	86.97±0.95	90.89±1.79	93.96±1.89	90.37±3.23	0.086
*C. neoformans*	***74.87±2.19 ***^***c***^	65.70±1.44	58.75±1.73	64.93±1.00	***0.024***
*T. marneffei*	82.02±0.52	118.58± 4.18	85.52±2.63	81.91±5.75	0.057

^**a**^The S/CO ratio was used to express antifungal antibody levels in different brands of IVIG. The average S/CO ratio value ± standard deviation of four fungal antigens in triplicate showed the differences among IVIG from four brands. Statistical analysis was performed by nonparametric Kruskal-Wallis test. ^**b, c**^ RAAS® IVIG had the highest level of antifungal IgG against *C. albicans* and *C. neoformans* among four brands

We compared different levels of antifungal IgG for each fungus using nonparametric Kruskal-Wallis test. The antifungal IgG levels among four brands of IVIG were statistically significant for *C. albicans* (*P* < 0.05) and *C. neoformans* (*P* < 0.05), while no significant differences for *A. fumigatus* (*P* = 0.086) and *T. marneffei* (*P* = 0.057) (Table 1). Group pairwise comparison was furthermore performed by independent samples t test among different brands of IVIG for specific IgG against *C. albicans* or *C. neoformans*. The highest level of antifungal IgG against *C. albicans* and *C. neoformans* was observed in RAAS® IVIG, while the lowest in Taibang® IVIG.

IVIG contained different levels of antifungal IgG specific for different fungi. When comparing the antibody levels for *C. albicans, A. fumigatus*, *C. neoformans* and *T. marneffei,* we noted IVIG contained significantly higher level of specific IgG for *C. albicans* than for other three fungi (*P* < 0.001) with the lowest for *C. neoformans.* No difference of the specific IgG level was observed between antifungal IgG specific *A. fumigatus* and *T. marneffei* (*P* = 0.772).

## Discussion

Humoral immunity plays important role in host against fungal infections, but little attention was paid on it. IgG against various beta-glucans including beta-glucans derived from *C. albicans,* laminarin, and pustulan were reported in normal human sera [10, 22]. Antifungal antibodies against *C. albicans* and *A. fumigatus* in normal human sera were sparkly reported in small number of samples [9, 11], which was consistent with the positive results of antifungal IgG in 563 normal human sera mixture. Further detection in several brands of IVIG derived from China were investigated and compared the antifungal IgG for both crude antigen and recombinant glycoproteins of *C. albicans*, *A. fumigatus*, *C. neoformans* and *T. marneffei*.

In this study, we found antifungal IgG against crude antigens of *C. albicans*, *A. fumigatus*, *C. neoformans* and *T. marneffei* were present in mixture of normal human sera from 563 healthy donors. Similar results were further confirmed in four brands of IVIG derived from China. Crude antigens of four clinical pathogenic fungi were observed with strong positive reaction with normal human sera and IVIG, among which *C. albicans* gave the highest positive signal. Mannan and beta-glucans were reported to be targets of antifungal antibodies against *C. albicans* [11, 22]. However, various antigens could attribute to heterogenic antibody response [23]. Beta-glucan which was abundant in most of fungi except for *C. neoformans* and *Mucor* species, reacted with specific IgG in normal human sera [22]. But reactions occurred between IVIG with *C. neoformans* (Fig. 1a, b) and *Mucor* (Data was not shown), which suggested antifungal IgG targets more antigens not just beta-glucans. Polysaccharides and glycoproteins are abundant in fungal cells, which could be expected the binding targets. Csa2, Afmp1cr, Cpl1 and Mp1p specific for four fungi were chose to verify the reaction with IVIG to different extent, of which Mp1p from *T. marneffei* reacts strongest than the others. Mp1p is a mannoprotein used for serodiagnosis for talaromycosis, which dampens host innate immune response [24]. Moreover, the binding of IVIG with recombinant Mp1p was stronger than crude antigen of *T. marneffei*, while an opposite phenomenon was observed in other fungi, especially *C. albicans*. It was in accordance with the study that antibody responses to different forms of *A. fumigatus* antigens were widely distributed in normal sera [9].

The binding with recombinant glycoprotein of *T. marneffei* also confirmed the binding diversity of antifungal IgG. The direct evidences of antifungal IgG were provided by several inhibition tests. Both fungal antigens and rabbit antifungal PAbs specific for *C. albicans*, *A. fumigatus*, *C. neoformans* and *T. marneffei* were used as inhibitors with efficient inhibition to prove the presence of antifungal IgG against fungal crude antigens. To the specific fungal proteins, Mp1p in *T. marneffei* was expected to be a new target of antifungal antibody in IVIG, according to results of inhibition tests and preabsorption by magnetic beads. Other glycoprotein, such Csa2 in *C. albicans* and Cpl1 in *C. neoformans* was probable target, with lower inhibitionthan Mp1p. However, whether antifungal IgG against Afmp1cr present in IVIG was not sure, according to the results of PAbs inhibition test, which was out of expectation. Although Afmp1p was a homolog of Mp1p [25, 26], antifungal IgG against Mp1p was confirmed to be present in IVIG, while anti-Afmp1cr antibodies was not found. It could be considered that Afmp1cr only as a part of Afmp1p [17]，may not be a predominant epitope.

To determine the difference among manufactures of IVIG, four brands of IVIG derived from healthy population from North (Taibang® IVIG), East (RAAS® IVIG), Middle (Nanyue® IVIG) and South (Intragam® IVIG) of China were tested. Significant difference of specific IgG against crude antigens of *C. albicans* and *C. neoformans* occurred among four brands of IVIG with no significant difference for *A. fumigatus* and *T. marneffei*. The discrepancy among IVIG from different manufactures may result from different procedures of preparations or sources of healthy populations. We noted that IVIG contained higher level of specific IgG for *C. albicans,* with low level for *A. fumigatus*, *C. neoformans* and *T. marneffei.* An interesting result showed that specific IgG for *A. fumigatus* showed no significant difference with for *T. marneffei*, a fungus endemic in Southern China. This finding suggested that antifungal antibodies in IVIG may be elicited by conserved antigens, including proteins and polysaccharides. It’s reported that antibodies generated against conserved bacterial polysaccharides were reactive with *A. fumigatus* [27].

The production of antifungal IgG in IVIG was probably related with fungal colonization in lung, gastric intestinal tract. In this study, antifungal IgG against *C. albicans* was found in normal human sera and IVIG, which may associate with colonization of *Candida*. As the most common commensal fungi in human, antibody against *C. albicans* was found in normal human sera and rodent model with *C. albicans* colonization [28, 29]. The antibody responses for *C. albicans* were shaped by commensal fungi in the human gut [30]. Not just in adulthood, naturally acquire antibodies against *C. albicans* and *C. neoformans* were developed by infancy [31]. This implied that the measurement of antifungal IgG could provide a screening test for antifungal humoral response, even for humoral immunodeficiency. *A. fumigatus* and *C. neoformans* widely distribute in the environment [32, 33], which could induce humoral response by inhalation of fungal cells. It is noteworthy that no significant difference among four brands of IVIG was found for antifungal IgG against *T. marneffei* derived from endemic and non-endemic areas. Intragam® IVIG derived from Hong Kong, an endemic area of talaromycosis, had no more antifungal IgG than others. However, colonization of *T. marneffei* was rarely reported. *T. marneffei* was formerly classified to *Penicillium*, which was ubiquitous airborne fungus in environment [33] and recognized as contaminating fungus. The specific IgG against *T. marneffei* may be induced by inhalation of *Penicillium* spores.

The role of antifungal antibodies in IVIG was not known. Antifungal antibodies against fungal glycoprotein or polysaccharides were reported to protect against murine models of aspergillosis, histoplasmosis, candidiasis and cryptococcosis [34–36], which provide a promising therapeutic option for refractory fungal infections. Recently, it was reported that the protective antifungal IgG could be induced by human gut commensal fungi and protect the host from systemic candidiasis [37].

Concerning these evidences of specific antifungal antibody present in IVIG, it would be important for understanding humoral defense against fungi. First, it may provide a basic perception of antifungal humoral immunity in Chinese normal adult population, which could contribute to the surveillance of humoral antifungal response in humans. Second, IVIG also could be expected to provide a novel therapeutic intervention for fungal infections. Successful treatment in chronic oral candidiasis patients with human polyvalent IgG [10], demonstrated the benefits of IVIG in fighting fungal infection. Further investigation on the role of antifungal antibody in IVIG should be performed. Third, the presence of antifungal antibody might lead to false negative results in antigen detection, which implied the importance of dissociation of antigen-antibody complex. This was consistent with the purpose of pretreatment of sera sample during galactomannan antigen detection [38].

Overall, this study provide evidence for antifungal specificity of IVIG from China. However, the health impact of antifungal antibody remains unclear and further investigations are needed. First, there would be further observation and research on the effect and mechanism of antifungal antibodies on fungi *in vivo* and *in vitro*. Second, IVIG would be expected to be an evaluation tool for humoral immunity against fungus, and also to be a possible choice for therapy of invasive fungal diseases.
